# A Pregnancy Case of Primary Mediastinal Large B Cell Lymphoma with Superior Vena Cava Syndrome

**DOI:** 10.1155/2021/3438230

**Published:** 2021-12-22

**Authors:** Lauren Brownhalls, Ann Gillett, Yasmin Whately, Keisuke Tanaka

**Affiliations:** ^1^Women's and Newborn Services, Royal Brisbane and Women's Hospital, Herston, Australia; ^2^Department of Hematology, Royal Brisbane and Women's Hospital, Herston, Australia; ^3^Department of Anaesthesia, Royal Brisbane and Women's Hospital, Herston, Australia; ^4^Faculty of Medicine, The University of Queensland, Herston, Australia

## Abstract

Primary mediastinal large B cell lymphoma (PMLBCL) is a subtype of non-Hodgkin's lymphoma which presents rarely in pregnancy. It is an aggressive tumour that is associated with symptoms of superior vena cava (SVC) compression and airway compromise such as dyspnoea, facial and arm swelling, cough, or chest pain. Timely diagnosis is imperative to optimising patient outcomes and reducing both maternal and fetal morbidity and mortality. We report a case of a 33-year-old woman diagnosed with PMLBCL who presented at 33-week gestation with SVC obstruction to 1 mm in diameter. After multidisciplinary team discussion regarding maternal and fetal implications of management options, we proceeded to a caesarean section and initiated chemotherapy postdelivery. Lower segment caesarean section was uncomplicated, and she underwent a cycle of R-CHOEP followed by 5 cycles of DA-EPOCH. Eighteen months since the completion of the chemotherapy, the disease remained in remission.

## 1. Introduction

Lymphomas represent a heterogenous group of lymphoid malignancies which differ in clinical presentation and treatment response. The World Health Organisation (WHO) classification system of lymphomas is based on cell type and separates lymphoid neoplasms derived from precursor lymphoid cells and mature lymphoid cells [1]. Non-Hodgkin's lymphoma (NHL) is a family of lymphoid malignancies derived from B and T cell progenitors, mature B and T cells, or natural killer cells. It is rare during pregnancy with an estimated incidence of 0.8 cases per 100,000 women [2].

Primary mediastinal large B cell lymphoma (PMLBCL) is classified as a distinct subtype of diffuse large B cell lymphoma [3]. The disease usually presents as a bulky mass in the mediastinum and occurs in approximately 2.5% of all non-Hodgkin's lymphoma [4]. The tumour consists of large cells with variable nuclear features, and diagnosis is made upon pathological evaluation of tumour tissue in the context of clinical findings [5, 6].

The clinical presentation of a locally invasive anterior mediastinal mass frequently involves airway compromise and superior vena cava (SVC) syndrome including dyspnoea, facial swelling, arm swelling, cough, or chest pain [7]. Given the limited literature of PMLBCL in pregnancy, the optimal management of these patients should be considered on a case-to-case scenario based on the expert advice of a multidisciplinary team. Primary management of these patients must weigh up the risks associated with premature delivery against the risks of initiating chemotherapy treatment in a pregnant patient.

We present a case of a woman presenting with symptoms of an SVC obstruction secondary to a PMLBCL in the third trimester who was delivered via caesarean section under combined spinal epidural anaesthesia, prior to initiation of chemotherapy treatment.

## 2. Case Presentation

**Figure 1:**
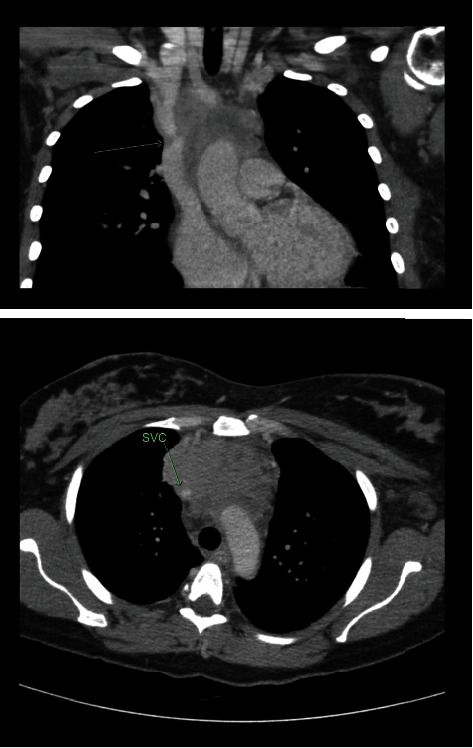
CT scan on presentation showing a mediastinal mass and concomitant obstruction of the SVC to 1 mm in diameter (green arrow).

A 33-year-old female, gravida 3 para 1 at 33 + 3-week gestation, presented with a five-day history of increasing bilateral upper limb swelling and orthopnoea. The pregnancy had been uncomplicated, and she had previously had one vaginal delivery at term. Chest computed tomography (CT) demonstrated a 92 × 50 × 64 mm soft tissue mediastinal mass obstructing the SVC to 1 mm diameter (Figure 1).

She was transferred to the intensive care unit (ICU) at a tertiary referral hospital. On examination, she was tachypnoeic with a respiratory rate of 24 with oxygen saturations of 98% on room air. She had obvious plethora and swelling of the face and upper limbs. Normal fetal growth and wellbeing were confirmed with cardiotocography and ultrasound scan.

The patient was reviewed by obstetric, anaesthetic, haematology-oncology, obstetric medicine, and cardiothoracic units. The primary concern for this patient was the critical SVC obstruction and associated risk of cardiovascular collapse. Antenatal corticosteroids were given, and she was commenced on eight-hourly doses of 8 mg of intravenous dexamethasone with the objective of reducing the size of the mediastinal mass. CT-guided biopsy was also performed upon start of the steroid therapy, and histopathology showed PMLBCL. Facial and upper limb swelling improved with dexamethasone. However, a repeat CT five days later revealed that the mass and the SVC obstruction remained unchanged and in addition, there was a thrombus in the left brachiocephalic vein extending into the left subclavian vein for which therapeutic enoxaparin was commenced.

Ongoing management plans were discussed at a multidisciplinary meeting. Commencement of antenatal chemotherapy was rejected due to the risk of fluid overload with intravenous fluid requirement in the setting of the major SVC obstruction. Decision was made to proceed to a caesarean section and to initiate chemotherapy treatment postdelivery.

Lower segment caesarean section was performed at 34 + 4-week gestation under regional anaesthesia with a slowly titrated combined spinal epidural. Advanced haemodynamic monitoring was used including intra-arterial line and cardiac output monitoring (FloTrac system®, Edwards Lifesciences, Irvine, CA, USA). In the event of cardiopulmonary collapse, emergent intubation and extracorporeal membrane oxygenation were planned. She delivered a live female baby weighing 2356 g with an APGAR score of 9 at 1 and 5 minutes, who required a 10-day stay in the neonatal intensive care unit.

The patient was admitted to the ICU postoperatively for observation given the concerns regarding postpartum fluid shifts. Her postpartum course was uncomplicated, and she was discharged from the ICU after 24 hours. Chemotherapy was commenced one week postpartum with a single cycle of rituximab, cyclophosphamide, doxorubicin, etoposide, vincristine, and prednisone (R-CHOEP) followed by 5 courses of dose-adjusted etoposide, vincristine, doxorubicin, cyclophosphamide, and prednisolone (DA-EPOCH) regimen.

**Figure 2:**
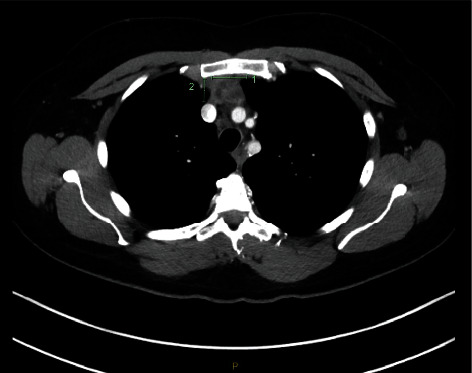
CT scan postchemotherapy treatment showing a reduction in the size of the mediastinal mass.

6 months after the delivery, CT scan showed a marked reduction in the size of the anterior mediastinal mass measuring 27 × 18 × 31 mm (Figure 2). She remained under the surveillance of the haematology-oncology team and was asymptomatic 18 months since the completion of the chemotherapy, and the disease was in remission.

## 3. Discussion

Case studies relating to anterior mediastinal masses have reported that patient's initial clinical presentation frequently involved symptoms such as dyspnoea, chest pain, palpitations, dry cough, and upper limb swelling [8–10]. Dyspnoea is a common presentation in pregnancy and is most commonly attributable to the normal physiological changes of pregnancy. Maintaining a level of clinical suspicion towards patients presenting with dyspnoea, in the context of other symptoms of airway compromise or SVC syndrome, is imperative to identifying those women who require further investigation and exclusion of an anterior mediastinal mass.

The majority of cases of PMLBCL in pregnancy were diagnosed at or prior to 30-week gestation and received primary chemotherapy treatment and delayed delivery [8]. Although initiation of antenatal chemotherapy was considered for our case, decision was made for delivery followed by postpartum chemotherapy. The risk associated with congestion secondary to two to three litres of intravenous fluid requirement daily for chemotherapy was deemed unacceptable.

Mode and timing of delivery for pregnant women with PMLBCL should also be based on multidisciplinary consensus considering gestational age, degree of SVC/airway obstruction, and administration of primary chemotherapy treatment. It was suggested that delivery should be timed 2-3 weeks after the most recent administration of chemotherapy in order for the mother and fetus to excrete chemotherapeutic agents [8]. A review of 12 cases of PMLBCL in the literature reported that 8 women delivered via caesarean section between 30- and 36-week gestation and 4 women had vaginal deliveries following induction of labour between 34- and 36-week gestation [8]. All 4 patients had undergone antenatal chemotherapy treatment, and their deliveries were reported as uncomplicated with all neonates healthy at short-term follow-up.

Patients with anterior mediastinal masses require careful perioperative planning given the risk of catastrophic airway or cardiovascular compression under general anaesthesia. Several case reports describe successful caesarean section in the parturient with such masses under combined spinal and epidural anaesthesia [11, 12]. In this case, near complete SVC obstruction required careful haemodynamic management due to poor tolerance of either blood loss or increased intravascular fluid volume.

Whilst DA-EPOCH is the standard chemotherapy regimen for PMBCL in our institution, this requires a central venous access device to be delivered. Given the critical SVC obstruction in this case, the probability of safely and successfully placing a central line was determined to be low and a single cycle of R-CHOEP delivered via a peripheral IV cannula was administered to debulk the tumour. The patient subsequently had a peripherally inserted central catheter (PICC) line placed successfully following SVC recanalization and was able to continue treatment with DA-EPOCH.

There were two reports of postpartum maternal deaths with advanced-stage metastatic disease. One presented with dyspnoea at 33 weeks and delivered by caesarean section. Following delivery, she underwent surgical debulking and received one cycle of chemotherapy prior to her demise at one-month partum [8]. The other woman presented at 30 weeks with progressive cardiac failure. She demised 8 months postpartum after 6 cycles of chemotherapy [9].

In summary, this was a rare case of PMLBCL with severe SVC obstruction in the third trimester that was successfully managed with caesarean section under combined spinal epidural anaesthesia and postpartum chemotherapy.
